# Future N deposition and precipitation changes will be beneficial for the growth of *Haloxylon ammodendron* in Gurbantunggut Desert, northwest China

**DOI:** 10.1038/s41598-018-37245-8

**Published:** 2019-03-08

**Authors:** Wen-Qin Zhao, Xin-hua Lv, Yong-guan Li, Zhong-ke Wang, Wei Zhang, Li Zhuang

**Affiliations:** College of Life Sciences, Shihezi University, Shihezi, 832000 China

## Abstract

Evaluation of precipitation and nitrogen (N) deposition in desert ecosystems helps to elucidate the reaction of desert ecosystems to future environmental changes. An *in-situ* field experiment was established to examine the influence of a long-term enhanced precipitation and N deposition on the photosynthetic traits and physiological characteristics of *Haloxylon ammodendron* in the Gurbantunggut Desert, northwest China, throughout the growing season in 2014–2016. Results showed a significant interaction between precipitation and N applications. Increased precipitation and N deposition and their coupling could significantly improve photosynthetic capacity, alter the variability in amplitude of water potential and change the content of substances regulating osmotic pressure in *H. ammodendron*. According to the comprehensive evaluation of *H. ammodendron’s* adaptability using six different water and N coupling models, a combination of a 30% increase in precipitation and a 30 kg N ha^−1^ yr^−1^ addition in nitrogen deposition, or the addition of N at a concentration of 60 kg N ha^−1^ yr^−1^ with natural precipitation were beneficial to *H. ammodendron* growth and development. Hence, changes in the future global environment can be anticipated to be beneficial to *H. ammodendron* growth.

## Introduction

Anthropogenic activity and climate change impacts on regional and global environments have been a feature during recent decades, including wide-ranging changes to patterns of precipitation and nitrogen (N) deposition^1^. The predicted increases in the rate of precipitation and N deposition for northwestern China by 2030 are 30%^2^ and 2.5 g N m^−2^ yr^−1^ ^3^, respectively. Future changes in N deposition and precipitation are expected to alter the function and structure of ecosystems^4,5^. Arid and semiarid areas are the most sensitive ecosystems to global changes, and their responses in hydrological, biological and biogeochemical respects are the most difficult to predict^6^. In particular, we do not know whether the response of desert ecosystems in northwestern China to these changes will be negative or positive.

In arid and semi-arid ecosystems, the availabilities of N and water are the major factors limiting plant growth, functional group-level community structure and composition^4,5,7,8^. Therefore, a small increase in precipitation and/or N deposition can have a disproportionately large ecological effect, and the contribution to plant yields within arid and semi-arid regions is much higher than that within other ecosystems characterized by high plant productivity^9^. Interactions between the effects of water and N addition on net primary production (NPP) and photosynthesis of desert plants^10^, physical and chemical properties of soil^11^ and soil microbial community structure^12,13^ have also been reported. However, once the precipitation and N inputs exceed biotic demands, the pulsed precipitation and additional N could have an adverse impact on the ecosystem, i.e., excess N could be exported from desert ecosystems through many pathways (erosion, soil gaseous emissions, leaching and greenhouse gas emissions)^14–16^, which could cause ecosystem nutrient imbalances^9,17^. The effects of pulsed precipitation could include gaseous N losses, ammonia volatilization and leaching in arid ecosystems^18^. Although low plant productivity is normal for (semi) arid ecosystems, these ecosystems cover a large proportion of the surface of earth^19^. Therefore, it is of great significance to evaluate the adaptability of plant responses to climate change, particularly changes in precipitation and N deposition in desert ecosystems. Desert vegetation responses to simultaneous increases in water and N have been widely reported; however, most of the research focused on plant morphological characteristics^5,20,21^, and less attention has been focused on physiological characteristics, particularly the shrub layer. As a shrub species that dominates single or mixed plant communities in the Gurbantunggut Desert, *Haloxylon ammodendron* is important for sand dune stabilization, and facilitating favorable nutrient and water conditions for other undergrowth vegetation, thereby maintaining the function and structure of arid ecosystems. Previous studies on *H. ammodendron* responses to global change have mainly focused on the seed-germination stage^22,23^, rhizosphere respiration^24^, community distribution and diversity^25,26^. However, relatively little attention has been paid to photosynthetic physiological responses to precipitation and N deposition and their combined effect, particularly the evaluation of physiological adaptability.

Recently, increased attention has been paid to identification of plant adaptability (stress resistance). Morphological, physiological, yield and ecological indices have been extensively applied to evaluate tolerance to stress^27–29^. However, plant adaptability is a complex process, and data gained from the use of a single indicator of the adaptability of plants can be influenced by the outside environment and variances in plant genotype, resulting in unreliable results. Thus, it is necessary to adopted a comprehensive multi-index evaluation method to determine the stress resistance of tested varieties^27,30^.

The present study used *H. ammodendron* of the southern Gurbantunggut Desert to investigate changes to physiological and photosynthetic characteristics of this species in typical desert regions of China under different precipitation and N conditions. Different multiple analysis methods were adopted for evaluating the adaptability of *H. ammodendron* through investigation of diurnal variation in photosynthetic gas exchange parameters and water potential and osmotic adjustment parameters. The relationship between physiological indices and adaptability is discussed, which provides the theoretical basis for the scientific management of desert ecosystems under global environmental change. Considering the potential for increased variability in nitrogen deposition and precipitation and a strategy of restoring desert ecosystems through establishing *H. ammodendron* forests, the objective of the present study were: (1) to understand the physiological responses of *H. ammodendron* to environment changes and; (2) to identify the optimal strategy for addition of water and nitrogen under current management of desert *H. ammodendron*. We initially hypothesized that: (1) increased precipitation would increase photosynthetic capacity and water potential, thereby reducing the content of osmotic substances, and; (2) the coupling model *W*_1_*N*_2_ is optimal for comprehensive physiological adaptability of *H. ammodendron* under the six different water and N coupling models.

## Results and Analysis

### Selection of evaluation parameters

**Figure 1:**
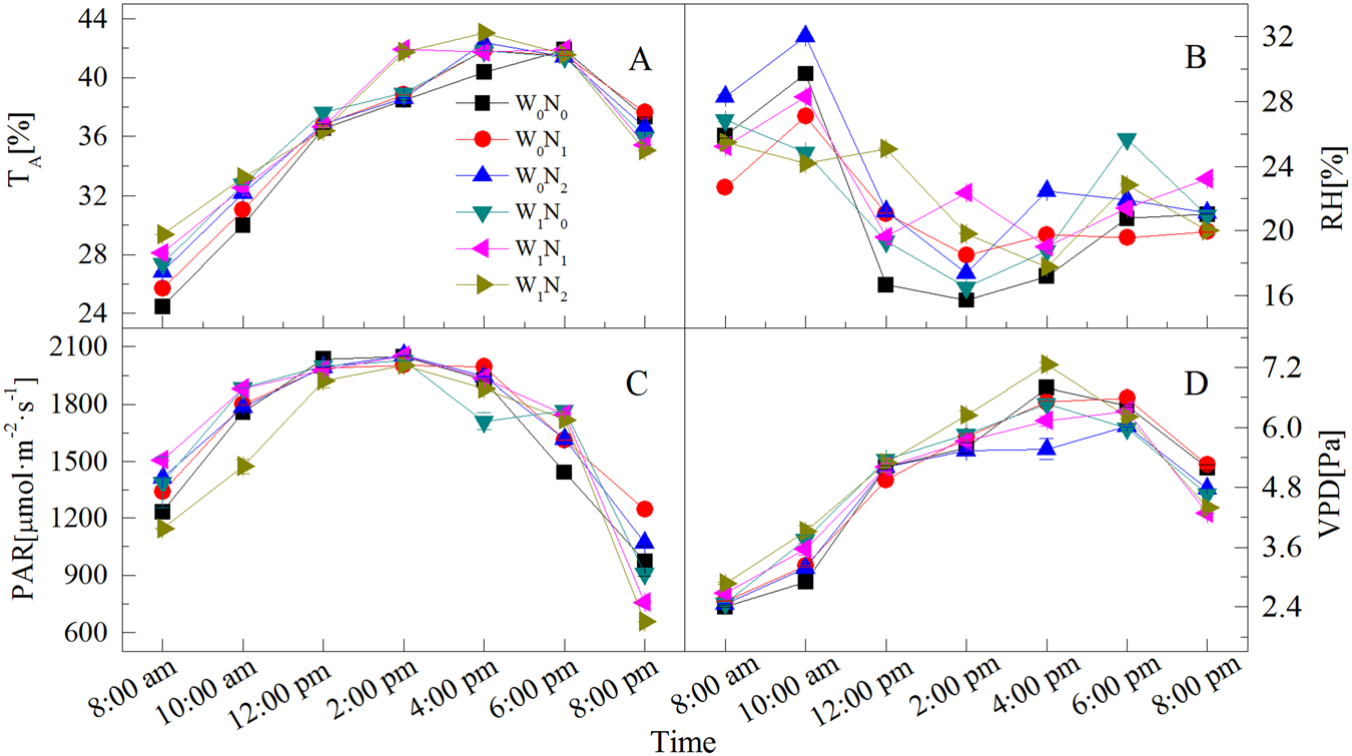
Diurnal variations of environmental factors under different water and N additions. (**A**) atmospheric temperature (*T*_*A*_); (**B**) relative humidity (*RH*); (**C**) photosynthetically available radiation (*PAR*); (**D**) vapor pressure deficit (VPD).

**Figure 2:**
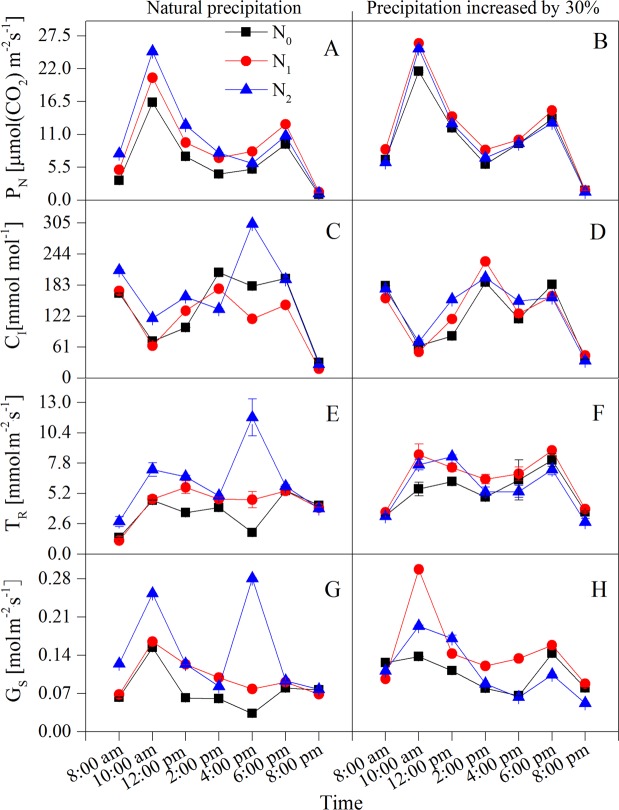
Diurnal variations of intercellular CO_2_ concentration (*C*_*I*_), net photosynthetic rate (*P*_*N*_), stomatal conductance (*G*_*S*_) and transpiration rate (*T*_*R*_) of *Haloxylon ammodendron* under water and N increased by 30%, respectively. (**A**,**B**) *P*_*N*_; (**C**,**D**) *C*_*I*_; (**E**,**F**) *T*_*R*_; (**G**,**H**) *G*_*S*_.

**Figure 3:**
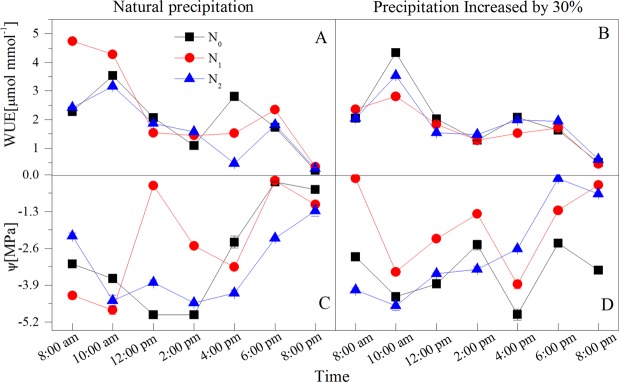
Diurnal variations of water potential (*Ψs*) and water use efficiency (*WUE*) of *Haloxylon ammodendron* under water and N increases of 30%, respectively. (**A**,**B**) *WUE*; (**C**,**D**) *Ψs*.

**Figure 4:**
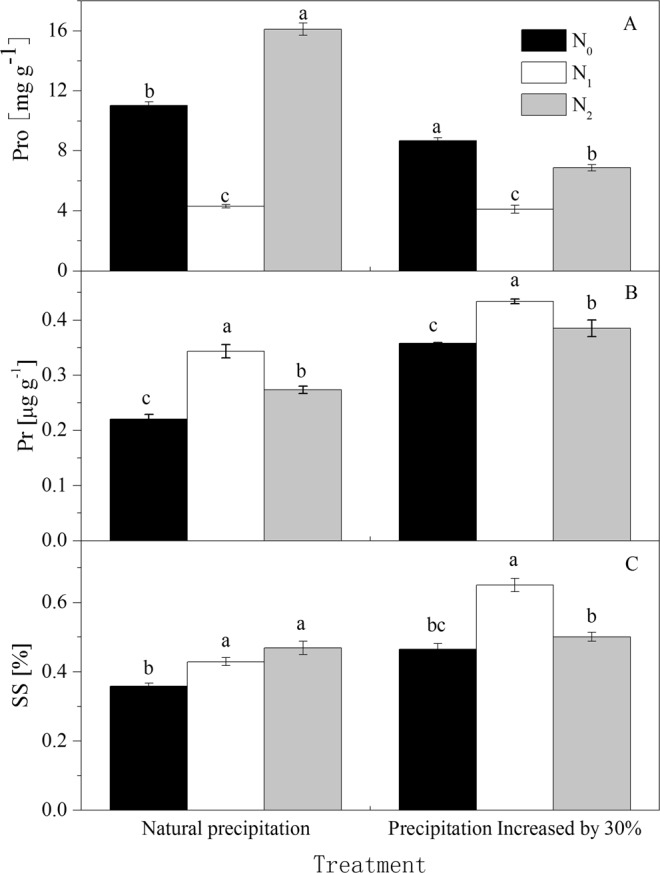
Effect of water and N addition on proline (*Pro*), soluble sugar (*SS*) and soluble protein (*Pr*) of *Haloxylon ammodendron*. Error bars represent standard deviation (n = 3). The letters placed above the bars indicate significant difference at P = 0.05. (**A**) *Pro*; (**B**) *Pr*; (**C**) *SS*.

The extreme diurnal variations of the environmental factors appear at 10:00 am and 2:00 pm (Fig. 1). The diurnal variation of photosynthetic parameters also showed that the intercellular CO_2_ concentration (*C*_*I*_), transpiration rate (*T*_*R*_), net photosynthetic rate (*P*_*N*_), water use efficiency (*WUE*) and stomatal conductance (*G*_*S*_) showed concurrent significant increases or decreases, as indicated by the maximum and minimum values in Figs 2 and 3. Water potential (*ψ*) measures during early morning (*ψ*_*e*_) reflected the recovery and water deficit of the plant, whereas the midday value (*ψ*_*m*_) reflected the maximum water deficit. The diurnal variation of water potential showed a maximum and minimum at 8:00 am (early morning) and 2:00 pm (midday), respectively. Hence, a total of 15 physiological indices were selected as individual indicators for comprehensive analysis: the time of 10:00 am (*T*_10_) and 2:00 pm (*T*_14_) measurements of *P*_*N*_, *T*_*R*_, *C*_*I*_, *G*_*S*_ and *WUE*, and the time of 8:00 am (*T*_8_) and the *T*_14_ measures of water potential and substances regulating osmotic pressure (proline, soluble sugar content, soluble protein content) (Fig. 4).

### Variance analysis of double factors

Variance analysis showed that there were significant differences among treatments for all indices (Table 1), except protein content in water and N coupling (*P* > 0.05). The results showed that the effects of precipitation and N deposition and their coupled effects on the physiological characteristics of *H. ammodendron* are positive.

**Table 1:** Two-way analysis of variance (ANOVA) analysis of physiological traits under water and N addition (F value).

Sources of variation	Precipitation	N addition	Precipitation * N addition
*T*_10_ *P*_*N*_	1965.965^***^	1803.995^***^	385.271^***^
*T*_14_ *P*_*N*_	108.24^***^	563.837^***^	122.792^***^
*T*_10_ *C*_*I*_	308.464^***^	295.349^***^	88.281^***^
*T*_14_ *C*_*I*_	653.73^***^	321.883^***^	417.629^***^
*T*_10_ *WUE*	114.528^***^	1011.485^***^	4067.736^***^
*T*_14_ *WUE*	23.197^***^	211.633^***^	58.144^***^
*ψ*_*e*_	440.289^***^	1576.57^***^	3722.973^***^
*ψ*_*m*_	281.726^***^	56.457^***^	150.78^***^
*T*_10_ *T*_*R*_	5673.968^***^	7749.193^***^	7147.501
*T*_14_ *T*_*R*_	2448.21^***^	2160.17^***^	1087.069^***^
*T*_10_ *G*_*S*_	575.893^***^	4899.633^***^	5570.136^***^
*T*_14_ *G*_*S*_	2289.487^***^	5413.773^***^	261.62^***^
*Pro*	700.816^***^	868.506^***^	330.002^***^
*SS*	178.154^***^	73.591^***^	34.448^***^
*Pr*	383.202^***^	96.322^***^	4.316

*** and ** indicates significant difference at the levels of p < 0.001 and 0.01, respectively.

*T*_10_ and *T*_14_ indicate the values measured at 10:00 am and 2:00 pm, respectively. *P*_*N*_: The net photosynthetic rate; *T*_*R*_: The transpiration rate; *Gs*: The stomata conductance; *WUE*: water use efficiency; *C*_*I*_: The intercellular CO_2_ concentration. The same definitions are applicable for the tables below.

### PAC and correlation analysis of various individual indicators

Calculations of the *PAC*s of photosynthetic parameters, water potential and regulators of osmotic pressure were based on the values obtained before and after water and N treatments (Table 2). We found that after the treatment, the *T*_10_*P*_*N*_, *T*_14_*P*_*N*_, *T*_14_*WUE*, *T*_10_*T*_*R*_, *T*_14_*T*_*R*_, *T*_14_*G*_*S*_, *SS*, *Pro*, *ψ*_*e*_ and *ψ*_*m*_ of assimilating shoots in different treatments were all enhanced (*PAC* > 1) compared with those of the control (*W*_0_*N*_0_), whereas the values of the other indices increased and decreased (*PAC* < 1) compared with those of the control with the addition of water and N (Table 2). Therefore, evaluating the adaptability of *H. ammodendron* to N and water addition in the southeastern Gurbantunggut Desert using only the *PAC* of one physiological index can lead to incorrect results.

**Table 2:** Physiological adaptability coefficient (*PAC*) of each index of the assimilating shoots of *Haloxylon ammodendron* under water and N addition.

water, N addition	T_10_P_N_	T_14_P_N_	T_10_C_I_	T_14_C_I_	T_10_WUE	T_14_WUE	ψ_e_	ψ_m_	T_10_T_R_	T_14_T_R_	T_10_G_S_	T_14_G_S_	Pro	SS	Pr
*W*_0_ *N*_0_	1	1	1	1	1	1	1	1	1	1	1	1	1	1	1
*W*_0_ *N*_1_	1.251	1.629	0.878	0.845	1.21	1.324	1.631	1.482	1.034	1.229	1.07	1.632	0.394	1.21	1.551
*W*_0_ *N*_2_	1.521	1.819	1.627	0.653	0.895	1.438	1.6	1.601	1.699	1.263	1.64	1.368	1.462	1.299	1.24
*W*_1_ *N*_0_	1.318	1.375	0.876	0.907	1.224	1.126	1.718	1.951	1.076	1.22	0.892	1.307	0.788	1.287	1.626
*W*_1_ *N*_1_	1.606	1.938	0.713	1.103	0.786	1.128	1.808	1.811	2.042	1.718	1.926	1.987	0.373	1.809	1.979
*W*_1_ *N*_2_	1.548	1.633	0.985	0.951	0.994	1.325	1.115	1.592	1.558	1.231	1.255	1.45	0.623	1.401	1.752

As can be seen from the correlation coefficient matrix of various treatments indicators (Table 3), varying degrees of correlation were evident among various indicators, suggesting overlaps in the information reflected. In addition, each index value provided a different input to the adaptability evaluation, i.e. one weight was associated with each index. As we know, adaptability in plants is a complicated process that is influenced not only by the plant genotype, but also by environmental conditions. Using only one index to evaluate adaptability therefore results in inaccurate information, and other multivariate statistical methods should rather be used.

**Table 3:** Correlation matrix of each index of the assimilating shoots of *Haloxylon ammodendron* under water and N addition.

Index	*T*_10_ *P*_*N*_	*T*_14_ *P*_*N*_	*T*_10_ *C*_*I*_	*T*_14_ *C*_*I*_	*T*_10_ *WUE*	*T*_14_ *WUE*	*ψ*_*e*_	*ψ*_*m*_	*T*_10_ *T*_*R*_	*T*_14_ *T*_*R*_	*T*_10_ *G*_*S*_	*T*_14_ *G*_*S*_	*Pro*	*SS*	*Pr*
*T*_10_ *P*_*N*_	1														
*T*_14_ *P*_*N*_	0.894**	1													
*T*_10_ *C*_*I*_	0.123	0.126	1												
*T*_14_ *C*_*I*_	−0.061	−0.181	−0.848**	1											
*T*_10_ *WUE*	−0.523**	−0.464**	−0.214	−0.216	1										
*T*_14_ *WUE*	0.537**	0.646**	0.603**	−0.761**	−0.025	1									
***ψ***_***e***_	0.487**	0.666**	−0.055	−0.218	0.049	0.293	1								
***ψ***_***m***_	0.764**	0.721**	−0.091	−0.134	0.033	0.427*	0.781**	1							
*T*_10_ *T*_*R*_	0.876**	0.807**	0.125	0.13	−0.858**	0.285	0.335	0.461*	1						
*T*_14_ *T*_*R*_	0.753**	0.797**	−0.323	0.365*	−0.564**	0.056	0.652**	0.621**	0.822**	1					
*T*_10_ *G*_*S*_	0.752**	0.800**	0.177	0.068	−0.882**	0.274	0.388*	0.336	0.956**	0.815**	1				
*T*_14_ *G*_*S*_	0.697**	0.833**	−0.396*	0.291	−0.351	0.222	0.663**	0.620**	0.671**	0.923**	0.683**	1			
*Pro*	−0.123	−0.203	0.887**	−0.673**	−0.174	0.227	−0.2	−0.252	−0.037	−0.460*	0.002	−0.649**	1		
*SS*	0.817**	0.769**	−0.321	0.387*	−0.562**	0.083	0.536**	0.666**	0.839**	0.943**	0.765**	0.855**	−0.431*	1	
*Pr*	0.698**	0.635**	−0.583**	0.443*	−0.129	0.087	0.506**	0.754**	0.527**	0.779**	0.389*	0.824**	−0.718**	0.855**	1

** and * indicate significant difference at the levels of p < 0.05 and 0.01, respectively.

### Principal component analysis

The *PAC* values of 15 individual indicators were calculated to minimize the overlap of information provided by indices, and these 15 physiological indices were used to develop three new indices. The first three indexes contributed 53.82%, 23.97% and 13.53%, respectively. The cumulative contribution of the first three comprehensive indices therefore reached 90.31%; hence, the contribution of the remaining indices is negligible and can be disregarded (Table 4). Based on individual contributions of each comprehensive index, their individual weights can be calculated.

**Table 4:** Coefficients of comprehensive indices [*Z*_*i*_] and proportion.

Principle factors	*Z*_1_	*Z*_2_	*Z*_3_
Eigen values	7.923	3.596	2.029
Contributive ratio	52.82%	23.97%	13.53%
Cumulative Contributive ratio	52.82%	76.79%	90.31%
Eigen vector *T1*0*P*_*N*_	0.111	0.088	0.026
*T14P*_*N*_	0.113	0.097	0.082
*T1*0*CI*	−0.027	0.27	−0.022
*T14CI*	0.029	−0.237	−0.225
*T1*0*WUE*	−0.069	−0.085	0.383
*T*1*4WUE*	0.034	0.203	0.213
*ψe*	0.082	0.006	0.247
*ψm*	0.086	−0.004	0.267
*T1*0*TR*	0.109	0.083	−0.189
*T14TR*	0.122	−0.038	−0.049
*T10GS*	0.103	0.095	−0.208
*T14GS*	0.116	−0.054	0.055
*Pro*	−0.052	0.221	−0.093
*SS*	0.121	−0.037	−0.051
*Pr*	0.105	−0.113	0.12

### Comprehensive evaluation of the adaptability of *H. ammodendron* under water and N treatments

#### Subordinate function analysis

Subordinate function values of various comprehensive indicators of each treatment were calculated in accordance with Equation (2) (Table 5) following PCA. For the same comprehensive indicator, such as *Z*1, under different water and N treatments, the maximum *u* (*X*_1_) was 1.000 for the *W*_1_*N*_*1*_ treatment and 0.000 for *W*_0_*N*_0_ (control). This suggested that when only *Z*1 was considered, *H. ammodendron* showed the highest level of adaptability to the *W*_1_*N*_*1*_ treatment, whereas its adaptability to the *W*_0_*N*_0_ (control) was the lowest. The adaptabilities to the remaining treatments were sorted according to the value of *u* (*X*_*i*_).

**Table 5:** The value of comprehensive index [*Z*_*i*_], index weight, *u*(*X*_*j*_), *D* value and comprehensive valuation for each treatment of water and N addition.

Water, N addition	*Z*_*1*_	*Z*_2_	*Z*_*3*_	u(X_1_)	u(X_2_)	u(X_3_)	D	Comprehensive comparison
*W*_*0*_ *N*_*0*_	−0.920	−0.118	−0.208	0.000	0.093	0.000	0.025	6
*W*_*0*_ *N*_1_	−0.123	−0.101	0.182	0.408	0.116	0.990	0.419	4
*W*_0_ *N*_*2*_	0.079	0.565	−0.006	0.512	1.000	0.514	0.642	1
*W*_1_ *N*_*0*_	−0.182	−0.158	0.184	0.378	0.041	1.001	0.382	5
*W*_1_ *N*_1_	1.031	−0.188	−0.130	1.000	0.000	0.198	0.614	2
*W*_*1*_ *N*_2_	0.114	−0.001	−0.023	0.530	0.248	0.473	0.447	3
Index weight				0.585	0.265	0.150		

#### Weight determination

Based on the contribution of various comprehensive indicators, the weights were calculated in accordance with Equation (3). The results showed that the three comprehensive indicators had weights of 0.585, 0.265 and 0.150, respectively (Table 5).

#### Comprehensive evaluation

The comprehensive physiological adaptability capabilities of *H. ammodendron* to various water and N treatments were calculated in accordance with Equation (4) (Table 5), and sorted based on the value of *D*. The adaptability order was: *W*_0_*N*_2_ > *W*_1_*N*_1_ > *W*_1_*N*_2_ > *W*_0_*N*_1_ > *W*_1_*N*_0_ > *W*_0_*N*_0_. To be specific, the minimum *D* value was obtained for *W*_0_*N*_0_, suggesting the lowest photosynthetic and physiological adaptability, whereas the maximum *D* value was for *W*_0_*N*_2_, suggesting the highest photosynthetic physiological adaptability. However, it is worth mentioning that the difference between *W*_0_*N*_2_ and *W*_1_*N*_1_ is not obvious; therefore, the photosynthetic physiological adaptability of *H. ammodendron* to two treatments was regarded as equal under different conditions.

## Discussion and Conclusion

As two main factors limiting desert ecosystems, studying the effects of N and water on desert plants can help us to understand the response by desert ecosystems to global changes. The additions of water and N are important means of regulating plant growth and development. Some previous studies have shown that water and N addition increases the productivity of plants by improving *WUE* and nitrogen-use efficiency (NUE) by increasing the rate of assimilation as a result of increased water and N investment in the photosynthetic apparatus and other biological processes^7,31–33^.

Many scholars have confirmed that increasing N fertilizer can improve the water utilization efficiency^34,35^, reflecting the coupling effect between N and water. Our results clearly showed that daily mean values of *P*_*N*_, *T*_*R*_, *G*_*S*_, *C*_*I*_ and *WUE* increased as water and N applications increased (Figs 1–4), and the photosynthetic capacity was significantly (*P* < 0.05) improved, which is consistent with our initial hypothesis. It should be noted that in *W*_1_ treatments, the maximum photosynthesis of the *N*_1_ level was obviously higher than that of *N*_2_, and the content of accumulated proline was the lowest and the plant could divert more energy to other physiological processes, indicating that the application of high nitrogen levels restrained photosynthesis to a certain extent and the N utilization efficiency of *H. ammodendron* decreased. However, under more arid conditions (in *W*_0_ treatments), the maximum photosynthesis of the *N*_2_ level was obviously higher than that of the *N*_1_ and *N*_0_ levels, and water potential gradually reduced, illustrating that the increased addition of N improves the photosynthetic rate and alleviates the pressure of environmental drought to a certain extent. The increased nitrogen concentration compensated for the plant damage caused by water deficit, since suitable application of N-fertilizers could raise water use efficiency, and further shows that the different water and N treatments influence plant water use efficiency differently^36^.

*H. ammodendron* showed obviously *P*_*N*_ midday depression in all treatments (Fig. 3A,B), possibly due to the extreme heat, drought, high light intensity, high evaporation and other harsh environmental conditions prevalent in the Gurbantunggut Desert. The present study estimated that the maximum value of *P*_*N*_ that appeared in *PAR* was 1762.11 mol m^−2^ s^−1^, whereas the *PAR* at 12:00 pm – 4:00 pm was significantly higher than that of the light saturation point of *H. ammodendron* (Fig. 1). In addition, the high temperature and low air humidity (*i.e*. high *VPD*) appear simultaneously at midday, which works against photosynthesis of *H. ammodendron*. Therefore, the decrease of the photosynthetic function under strong light conditions is a preservation reaction to escape heat and drought damage. According to the criteria proposed by Xu^37^, the changing direction of *C*_*I*_ alone could determine whether *P*_*N*_ is subject to stomatal limitation or non-stomatal limitation. In the present study, *C*_*I*_ in all treatments at midday except in *W*_0_*N*_2_, almost reached the maximum value, and *G*_*S*_ showed a sharp decline (Fig. 2). Therefore, the cause of the *P*_*N*_ midday depression in *H. ammodendron* can be attributed to the result of the combination of stomatal and non-stomatal (harsh environmental conditions) factors.

In the present study, PCA was adopted to transform 15 individual indicators of assimilating shoots under different increased precipitation and N treatments into three independent comprehensive indicators, thus obtaining the *D* values under different future changes. Subsequently, the comprehensive physiological adaptability capabilities under six water and N addition treatments were sorted based on the *D* value, and the order of adaptability was: *W*_0_*N*_2_ > *W*_1_*N*_1_ > *W*_1_*N*_2_ > *W*_0_*N*_1_ > *W*_1_*N*_0_ > *W*_0_*N*_0_. Thus, it is evident that the coupling effect of increased water and N is reflected in the physiological adaptation of *H. ammodendron*. In addition, precipitation and N deposition interactive responses of physiological effect were much lower than those of *N*_2_ single-factor, but still increased photosynthesis overall. And the *D* value of *W*_0_*N*_2_ was significantly higher than that of *W*_1_*N*_2_, which is not consistent with our original hypothesis, suggesting that the water and N coupling effects on *H. ammodendron* are biased towards the low N treatment, and the effect of “raising water use efficiency by suitable application of fertilizers”^38–40^ is obvious under certain water stress. It is important to note that the *D* values of *W*_1_*N*_1_ and *W*_0_*N*_2_ were 0.614 and 0.642, respectively, and the difference is not very obvious; therefore, they can be considered as equivalent to a certain extent. The significance of this is that to achieve the equivalent effect, i.e. more N is required without increasing water, or under increased precipitation, only small quantities of N fertilizer are required. It can be said that the two strategies are associated with highly effective use of water and fertilizer resources in arid areas. This demonstrates that *H. ammodendron* has evolved a unique physiological adaptation to water and N.

Water is a fundamental natural resource maintaining the ecology of the northwest arid region of China. The warming of the climate is exacerbating extreme hydrological events, which results in increased uncertainty in regards to water resources, the water cycle and the regulation of ecological water demand in the northwest arid region^41,42^. In the present study, the effects of two treatments (*W*_1_*N*_1_ and *W*_0_*N*_2_) on comprehensive physiological characteristics were shown to be suitable for countering the impact of the uncertainty associated with future water resources. Within desert ecosystem management, when water is abundant, the addition of a small amount of N can ensure the normal growth of *H. ammodendron*. In contrast, when water is scarce, more N is required to allow *H. ammodendron* to deal with drought stress. It must however also be considered that not only N, but also phosphorus (P) is an essential nutrient for plant growth. However, the present study focused on the influence of water and N deposition under the assumption of no P limitation to growth, and the analysis of the influence of P is left to future studies. The conclusion of the present study is consistent with the theoretical concepts of “raising water use efficiency by suitable application of fertilizers” and “promoting fertilizer use efficiency by suitable application of water”^38,39,43^, and suggests that increased precipitation and nitrogen deposition under future global environmental changes will be beneficial to the growth of desert plants. In addition, the optimal current models for improved management of desert *H. ammodendro*n are *W*_0_*N*_2_ and *W*_1_*N*_1_.

## Materials and Methods

### Study Site

Native habitats of *H. ammodendron* in the southeastern Gurbantunggut Desert (44°30′N, 87°45′E and 437.1 m a.s.l.), near the Fukang Station of Desert Ecology, Chinese Academy of Sciences, northwestern China, were chosen as the experimental sites, from April 2014 to October 2016. The region has a temperate arid continental climate with cold winters and dry hot summers, and the growing season is from April to October. The average annual precipitation and temperature are 215.6 mm (the precipitation during April to October accounted for 70–80% of the annual precipitation) and 7.1 °C (the monthly average temperature was highest in July at 33.6 °C and the lowest in January at −21.8 °C), respectively, with a potential evaporation of approximately 2,000 mm^14^. The dominant soil type is grey desert solonetz (pH = 9.55 ± 0.14) with a low fertility with aeolian sands on the surface (0 cm–100 cm). The total N content and ratio of C to N are 0.08 g kg^−1^ ± 0.003 g kg^−1^ and 21.39 ± 1.84, respectively^14^. *Haloxylon persicum* and *H. ammodendron* dominate floral species, with approximately 30% coverage^44^, with herbs comprising some other dominant species.

### Experiment design

The experiment simulated two levels of N deposition: (1) the existing N deposition rate of the Gurbantunggut Desert affected by pollution through fertilizer use, coal consumption, urban traffic and industrial development and with an average annual increase of approximately 30 kilograms of nitrogen per hectare (kg N ha^−1^ yr^−1^); (2) the rate predicted for the future (60 kg N ha^−1^ yr^−1^), based on the forecast of Galloway^45^ *et al*., who found that global N deposition will double by 2050 relative to the early 1990s. The treatment of additional water simulated an additional precipitation of 60 mm, as per the prediction by Liu^2^ *et al*. of a 30% increase in precipitation in this region in the next 30 years. During April 2014 to October 2016, an experiment was conducted *in-situ* to study the adaptability of *H. ammodendron* responses to a long-term (three-year) increase in N deposition and precipitation based on characteristics of physiological and photosynthetic. The complete block interactive field experiment consisted of two levels of water addition (*W*_0_: natural precipitation, control; *W*_1_: an increase of 30%, i.e., an additional 60 mm annual precipitation) and three levels of N addition (*N*_0_: 0 kg N ha^−1^ yr^−1^, control; *N*_1_: 30 kg N ha^−1^ yr^−1^, *N*_2_: 60 kg N ha^−1^ yr^−1^)^14^. Therefore, six experimental groups (*W*_0_*N*_0_, *W*_0_*N*_1_, *W*_0_*N*_2_, *W*_1_*N*_0_, *W*_1_*N*_1_ and *W*_1_*N*_2_) were tested with each treatment comprising four replicates. The plots each had an area of 100 m^2^, separated by a buffer zone of 5 m width. The average coverage and height of *H. ammodendron* were 1.93 m^2^ and 1.54 m, respectively, with no statistically significant differences across the plots evident when using the block as a covariance variable in analysis of covariance (ANCOVA) analysis. The additional precipitation and N deposition (NH_4_NO_3_) treatments were conducted in equal amounts over twelve applications, once a week in April, July and September during 2014 to 2016, equating to precipitation of 5 mm and 5 kg N ha^−1^ or 2.5 kg N ha^−1^ per application over a week. The NH_4_NO_3_ was diluted in 50 L water (equivalent to precipitation of 0.5 mm), and evenly applied after the simulated precipitation. Water and NH_4_NO_3_ was applied using a nebulizer, through irrigating the *H. ammodendron* canopy in the late afternoon or early morning to reduce evaporation. The same water volume was applied to the zero N treatments. Stainless steel sheeting was used as a border around all plots to reduce lateral water and N losses^14^. The photosynthetic gas exchange parameters, water potential and the content of substances regulating osmotic pressure were measured in three consecutive sunny days of early July 2016 (the period of vigorous growth of *H. ammodendron*).

## Experimental Methods

### Gas exchange parameters

Gas exchange for the annual assimilating shoots of *H. ammodendron* was determined. The diurnal variations of the rates of net photosynthesis (*P*_*N*_), transpiration (*T*_*R*_), stomatal conductance (*G*_*S*_) and concentrations of intercellular CO_2_ (*C*_*I*_) were measured, recorded and calculated simultaneously by a portable photosynthesis measurement system (LI-6400XT, LI-COR, Lincoln, NE, USA) every 2 h from 08:00 am to 8:00 pm on sunny days in July. A 2 cm × 3 cm chamber was used to measure the gas exchange of assimilating shoots. The efficiency of water use (*WUE*) was calculated using with the equation *WUE* = *P*_*N*_/*T*_*R*_. The diurnal variations of atmospheric temperature (*T*_*A*_), relative humidity (*RH*), vapor pressure deficit (*VPD*) and photosynthetically active radiation (*PAR*) were simultaneously measured. Three replicates were sampled from each individual plant, with the mean of each consecutive stable 10 data points calculated.

### Water potential

Assimilating shoot water potential (*ψ*) of *H. ammodendron* was measured using a HR-33T Dew Point Microvoltmeter (WESCOR, USA). The well-developed annual assimilating shoots of the trunk of *H. ammodendron* situated 1.3 m above the ground and directly exposed to sunlight were collected, and the middle section of the shoots was extracted and immediately placed in the C-52 sample chamber of a Dew Point Microvolt-meter. The sample chamber was connected to a water potential meter. The *μv* value was then measured and the potential value (*MPa*) of the shoot sample was calculated according to the formula *μv* = (dew-point value)/7.5. The photosynthetic parameters and the daily variation was simultaneously measured. The measurements of variation in water potential were conducted from 8:00 am to 8:00 pm and samples were collected every two hours. The process was repeated three times for calculation of a mean.

### Substances regulating osmotic pressure

Samples were collected from the same parts of assimilating shoots while simultaneously measuring photosynthetic parameters from 10:00 am to 12:00 pm. The samples were immediately stored in a liquid N tank for transportation back to the laboratory for measurement of regulators of osmotic pressure. The contents of relative soluble sugar (*SS*), proline (*Pro*) and soluble protein (*Pr*) were determined with the anthrone ethyl acetate, ninhydrin colorimetry and Coomassie Brilliant Blue staining methods, respectively^46^.

### Statistical analysis

Origin 8.0 for Windows (OriginLab Software Inc., CA, USA) and SPSS 19.0 statistical package (SPSS Inc., Chicago, IL, USA) software were adopted for principal component analysis (PCA), variance analysis and correlation analysis. Two-way analysis of variance (ANOVA), using the water and N conditions as two independent variables, was applied to determine the differences in parameters (gas exchange, water potential, *SS*, *Pro* and *Pr*) between treatments, and statistical significance was assumed if *P* < 0.05. The comparison of means among treatments was based on the least significant difference (LSD) test at the 5% probability level. An analysis of the relationships between parameters was conducted using the Pearson linear correlation and the correlations were built from the means of N and water measurements within each treatment.

Related indicators were calculated using a method adapted from Cao^27^ *et al*. The photosynthetic physiological adaptability coefficient (*PAC*) was calculated as:

1 $$PAC={\rm{Determined}}\,{\rm{value}}\,{\rm{of}}\,{\rm{treatment}}/{\rm{Determined}}\,{\rm{value}}\,{\rm{of}}\,{\rm{control}}$$

Subordinate function values of various comprehensive indicators of different treatments were calculated as:

2 $$u({X}_{i})=({X}_{i}-{X}_{{\min }})/({X}_{max}-{X}_{min})\,(i=1,\,2,\,\ldots ,\,n)$$

Weights of various comprehensive indicators were calculated as:

3 $${W}_{i}={P}_{i}/\sum _{i=1}^{n}Pi\,(i=1,\,2,\,\ldots ,\,n)$$

The *D* values of the different treatments were calculated as:

4 $$D=\sum _{i=1}^{n}[u(Xi)\times Wi]\,(i=1,\,2,\,\ldots ,\,n)$$

*X*_*i*_, *X*_*min*_, *X*_*max*_ and *W*_*ἰ*_ are the score, minimum score, maximum scores and importance (weight) of the ith comprehensive indicator, respectively; *P*_*i*_ is the contribution rate of the ἰth comprehensive indicator of various treatments of *H. ammodendron*; *D* is the comprehensive evaluation value for adaptability under different treatments of water and N.
